# Lymph Node Transplantation and Its Immunological Significance in Animal Models

**DOI:** 10.1155/2011/353510

**Published:** 2011-05-25

**Authors:** Manuela Buettner, Ulrike Bode

**Affiliations:** Institute of Functional and Applied Anatomy, Hannover Medical School, 30625 Hannover, Germany

## Abstract

Lymph nodes (LNs) are distributed all over the body and whatever the site consists of the same cell populations. However, there are great differences between LN from different draining areas. For example, in mesenteric LN, homing molecules, for example, CCR9 and **α**4**β**7 integrin, were induced and cytokines, for example, IL-4, were produced on higher levels compared to peripheral LN. To study the immunological functions of LN, LN transplantation was performed in some specific areas using different animal models. Many groups investigated not only the regeneration of transplanted LN but also the induction of immune responses or tolerance after transplantation. Existing differences between LNs were still detectable after transplantation. Most important, stromal cells of the LN were identified as responsible for these differences. They survive during regeneration and were shown to reconstruct not only the structure of the new LN but also the microenvironment.

## 1. Site-Specific Immunological Differences of Lymph Nodes

The primary, secondary, and—in some circumstances—tertiary lymphoid tissue together form the lymphoid system. The primary lymphoid organs are the bone marrow (BM) and the thymus, while the secondary lymphoid organs consist of the spleen, the Peyer's Plaques (PPs) and the lymph nodes (LNs). The formation and the development of tertiary lymphoid tissues only occur during inflammation and infection. Focusing in this paper on our and other studies concerning LN transplantation, all other lymphoid tissue structures will not be included (for more details see [1]).

LN are unique in morphology and function. They filter and scan the lymph for antigens. Recognizing a pathogenic antigen (Ag), an immune response is induced, whereas recognizing a harmless Ag, tolerance develops. However, there are great differences between LNs from different draining areas. Several years ago a different cytokine milieu was found in mesenteric lymph node (mLN) draining Ag from the gut, compared to peripheral lymph node (pLN) which drains the skin. The mRNA level of interleukin-4 (IL-4) and transforming growth factor-*β* (TGF*β*) was much higher in mLN, whereas IL-2 and interferon-*γ* (IFN*γ*) were decreased compared to pLN [2]. Immune cells from skin draining LN, for example, show other surface molecules in comparison with lymphocytes isolated from the gut draining LN. Lymphocytes of the mLN are imprinted to upregulate CCR9 and the adhesion molecule *α*4*β*7 integrin on their surface [3, 4]. The specific ligands of these molecules are also exclusively found in the gastrointestinal tract. The mucosal addressin cell adhesion molecule-1 (MAdCAM-1), the ligand for *α*4*β*7 integrin, is found on high endothelial venules (HEVs) in the gut and also in the mLN but could not be detected in pLN [5]. However, lymphocytes showing a skin-homing pattern are positive for E- and P-selectin ligands, and the chemokine receptor CCR4 is thought to be an additional skin-homing marker [6].

Furthermore, dendritic cells (DCs) coming from the gut have a unique phenotype. Most of them show a high expression of the major histocompatibility complex class II (MHCII) and are positive for CD103 and CD11c [7]. The enzyme retinal dehydrogenase 2 (RALDH2) is produced mainly on gut DC [8]. It was shown that RALDH2 is a metabolite in the oxidation of vitamin A to retinoic acid (RA), which is important for the induction of the gut-homing molecules (CCR9 and *α*4*β*7 integrin) on lymphocytes [3, 4]. By contrast, most of the DC in pLN are langerin positive but CD103 and RALDH2 negative [4, 9, 10]. There is great disparity in knowledge about LN: much is known about the mLN, such as activation and homing of lymphocytes and the presence of site-specific DC. In contrast, fewer details are known about pLN regarding their LN-specific expression while nothing is known about other LN, for example, the cervical LN (cLN) or the coeliac LN.

## 2. The Transplantation Model

**Figure 1:**
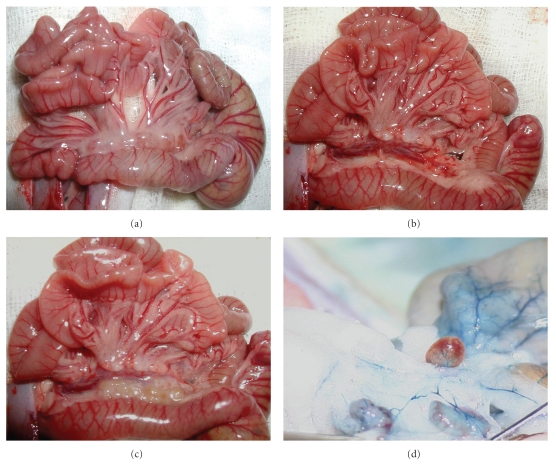
The gut system with mLN during operation and after regeneration. Rats were anesthetized and the abdomen was opened. The gut was taken out and the mLN were seen (a). The mLN were removed carefully (b). Afterwards the donor LN were placed in the vacant area (c). The gut was replaced in the abdomen and the abdomen closed. After 8 weeks LN were analyzed by injecting a dye (Berlin blue) which is transported via the lymphatics into the transplanted LN (d).

To study the immunological functions of mLN and pLN, we established a surgical technique, removing the mLN and transplanting a pLN or another mLN into the mesentery. This was done in rats as well as in mice. Briefly, the animals were anesthetized and the abdomen was opened. The gut was taken out so that the mLN were seen (Figure 1(a)). The mLN were removed carefully not injuring the blood vessels lying behind, whereas the connection of the lymph vessels to the LN was disturbed (Figure 1(b)). Previously excised mLN or pLN from a donor animal were transplanted into this vacant area (Figure 1(c)). After this, the gut was replaced carefully in the abdomen and the abdomen was closed. In rats, blood and lymph vessel connections were observed within two weeks (Figure 1(d)). 

There are many other draining areas in the body, and in some of them LN dissection followed by transplantation could be performed. For example, LN from the skin draining site, the auxiliary LN (axLN) [11], the popliteal LN (popLN) [12–16], or the inguinal LN (ingLN) [17–21] were removed by many groups, and different LNs (mLN, ingLN, and popLN) were transplanted into these areas. In addition, LNs were transplanted in the four mammary fat pads (MFP) or under the skin behind or at the ear without removing any LN [22–25]. Also, the LN, which drain the head neck region (cLN) were dissected and replaced by a cLN or mLN [26, 27]. In addition, mLN dissection with following LN transplantation (pLN, mLN) [28–31] was performed. Furthermore, the kidney capsule or the greater omentum was tested as an area of LN regeneration [20, 22, 32, 33].

For LN transplantation, different animal models are available. This technique has been used in rats [15, 19, 24, 28, 29, 32], mice [11–13, 26, 27, 29–31, 34], rabbits [25], pigs [17, 18, 20, 33], sheep [14], dogs [21] and for clinical applications also in humans [35, 36]. There has also been transplantation between different species, for example, human LNs were transplanted into immune-suppressed mice [22–24].

Most of the studies analyzed the structure of LN for a better understanding of the basic mechanisms during regeneration, although some studies focused on the function of LN, for example, immune response or tolerance induction. Only few studies were performed in respect to human diseases. One of them deals with graft versus host disease (GvHD), where the draining LN or in particular the specific microenvironment of the LN was found to be important [26, 37]. Another disease which was analyzed regarding LN transplantation is cancer, especially the role of the lymphatic system in tumor growth [11, 16, 24, 38]. Different tumor cells were injected into animals, and later on LN of the draining area were removed and transplanted into untreated animals to analyze the metastatic capacity of the tumor cells [24, 38]. Rabson et al. demonstrated the function of LN transplants mounting a cytotoxic response to tumor cells [16] and Tammela et al. identified transplanted LN as a barrier for metastatic tumor cells [11]. Thus, LN transplantation allows the characterization of tumor cell lines concerning their metastatic capacity. Furthermore, the role of the lymphatics and LN in cancer can be analyzed in more details.

The only disease to be analyzed using both basic experiments and involving clinical practice is lymphedema. After cancer therapy, a major problem for the patients is the development of a lymphedema. Lymphedema is the concentration of extracellular fluid, the accumulation of macromolecules and cells in the interstitium through the adynamic function of lymph fluid transport. Most studies aim to establish a therapy. LNs were transplanted into the edema region, and later on the accumulation of water was measured. Chen et al. demonstrated LN with normal architecture and size, three and six months after transplantation. Furthermore, they found a regenerated lymph system using lymphangiography which was already functional three months after transplantation and much improved six months later. The circumference of the limb was reduced after transplantation compared with preoperative data [21]. Similar results were observed by other groups in other animal models, for example, rabbits or sheep [14, 25]. A few clinical studies have been performed in the last few years. In these studies, an ingLN was transplanted in the axillary region after breast cancer therapy and lymphadenectomy, and several months later, the incidence of edema or neuropathic pain was determined. Positive and persistent effects were found not only regarding the improvement of the lymphedema but also neuropathic pain. Thus, LN transplantation seems to be a good therapeutic approach [35, 36].

## 3. LN Transplantation Shows the Function of LN Regeneration

The regeneration of LN is a major aspect after transplantation. In the early years of establishing LN transplantation, different areas were tested, for example, sites where splenic transplantation had previously succeeded. After transplantation of LN into the greater omentum, no regenerated LN could be found whereas the kidney capsule was found to be a better transplantation site [20, 32, 33]. However, different areas in the skin were shown to be the most suitable places for LN transplantation [20, 33]. Although mLN transplantation in the mesentery was performed in the early years, regeneration of the transplanted LN could not be detected [33], although in later studies we and others found regenerated LN in this area [28–30].

Furthermore, the development of the regeneration of LN was documented. In rats, Liu et al. showed one month after transplantation reduced re-circulating lymphocytes, decreased or disappeared germinal centers and follicles, and less prominent HEV, but two months later normal compartment structures in a transplanted mLN [28]. In addition, the compartment structure and also the cell subset composition of a human LN transplanted into immune-suppressed mice normalized three months after transplantation; however, no germinal centers were identified [22].

**Figure 2:**
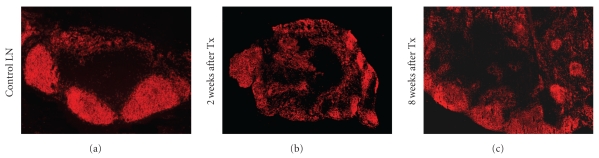
Early after regeneration LN are disorganized whereas after two months LN are regenerated. Cryosections of transplanted LN were stained with mAbs against B cells. The mLN of an untreated animal shows a typical compartmental structure, whereas two weeks after transplantation the architecture of the compartments is destroyed. Only small clusters of B cells are seen. Eight weeks after transplantation large B cell areas are again found, comparable to the control mLN.

We also analyzed the regeneration of the transplanted LN (LNtx). The mLN were removed, and an mLN as control or a pLN was transplanted. To identify the regeneration of LNtx, a kinetic study was performed and the architecture of the LNtx was analyzed (Figure 2). During the first weeks, a disturbed LN was found with disorganized compartments for B and T cells [29]. Over a period of eight weeks, a fully regenerated LN developed with connections to lymph and blood vessels [29]. All vessels were shown to be functional, transporting lymph fluid or lymphocytes into the transplanted LN. Furthermore, we could clearly show that lymphocytes from the donor LN disappeared from the LNtx and lymphocytes from the recipient repopulated the transplanted LN. In addition, DC from the draining area migrated from the gut via the afferent lymphatics into the LNtx. Over the whole period of examination, CD103^+^ DCs were identified in pLNtx [9, 29]. 

Furthermore, using transgenic mice which express the GFP gene in all cells, we showed for the first time that stromal cells remained in the tissue during regeneration [29, 30]. Stromal cells are nonhematopoietic cells, which form the skeletal backbone of the LN by forming a network and extracellular matrix components [39, 40]. Most of these stromal cells could be identified as GFP positive, even eight weeks after transplantation [29]. Different stromal cell subpopulations were identified. The two major subpopulations are fibroblastic reticular cells (FRC) and follicular dendritic cells (FDCs) which are accompanied by endothelial cells from lymph and blood vessels. We found that most FRCs and endothelial cells are GFP positive and therefore remain in the transplanted LN, whereas only half of the transplanted FDC could still be detected after regeneration. Furthermore, we recently demonstrated that stromal cells are not only necessary but also responsible for a successful regeneration of LN by mediating chemokines such as CXCL13 or CCL21/CCL19 (Buettner et al. [41]).

## 4. Connection to Blood and Lymph Vessels

The vascular requirements for the regeneration of LN were analyzed. LN of rats were transplanted into the popliteal fossa with microsurgical anastomosis, with vascular pedicles or with no vascularization. Functional vessels and normal histology were identified in the transplants with anastomosis, whereas LN with no vascularization underwent fibrosis and were not detectable via radioactivity within six weeks [15, 16]. However, in the canine model, Chen et al. observed no difference in regeneration after three months between the anastomotic group and the spontaneous reconnection group [21]. In addition, we and many other groups transplanted LN into different regions of rats and mice with no vascularizations, and subsequently regenerated LNs were detected [13, 27, 29]. These differences are hard to explain, but may be due to the different animal models, transplantation sites or regeneration times. For a successful regeneration of LN the decisive factors are probably the site of transplantation, the type and origin of LN and the time of regeneration.

HEV were extensively studied after transplantation. In our transplantation model, we were able to identify functional HEV two weeks after transplantation, thereby detecting previously injected lymphocytes within the HEV of transplanted LN [29]. Mebius et al. also found reconnected HEV early after transplantation [12]. Sasaki et al. studied the reconnection of HEV in more details. They found that capillaries started to invade the LN three days after transplantation, and after day five these sprouted into the graft. HEV appeared ten days after transplantation and the subcapsular sinus was formed. From day 28, the transplanted LN were structurally complete although no germinal centers were seen [32]. After transplantation of a human LN into immune-suppressed mice, Blades et al. found both human and murine vessels to be present and still functional in the transplant, but the cellularity and organization of LN were reduced compared with the original LN [23]. Thus, the connection of the blood vessels to transplanted LN has been a topic of great debate and seems to be dependent on the method of transplantation.

Fewer differences between the various studies were found in the detection of the connection of lymph vessels. We identified the connection of the LN to the draining area by injecting a dye which is transported only via the lymphatics into the LNtx (Figure 1(d)). By applying oil by oral gavage we showed the LNtx connection to the lymphatic vessels in mice much more easily. The oil is transported by the lymphatic system whereby the lymph system appears white. To identify lymph vessel connection after LNtx in regions other than the gut, for example, the skin, a dye can be injected into the draining area which is then transported via the lymph vessels. A more technical version for high-resolution analysis is lymphangiography or lymphoscintigraphy (2D methods) or SPECT-CT or emission computerized tomographic scanning (3D techniques), in which contrast medium is injected and the lymph vessels are highlighted [11, 16, 17, 21, 42]. These techniques allowed a series of scans in animals or humans to study the lymphangiogenesis and the connection of lymph vessels to the transplanted lymph node in vivo.

## 5. Further Basic Studies

The time point of transplantation was studied to identify the best conditions for LN regeneration. On the one hand, the effect of the age of the recipients on the regeneration of LN was explored. Regenerated LNs were always found, showing no difference between young and older animals [20]. On the other hand, the reconnection of lymph vessels to transplanted LN was analyzed after direct transplantation, or with an interval of two or seven days between dissection and transplantation. Fewer reconnected lymph vessels were found in animals in which transplantation was delayed [15]. In addition, Becker et al. looked at edema patients and found the best results when the duration of edema was shortest before transplantation [35].

Another question in the last few years was how to speed up or enhance the regeneration of LN. Several agents were applied, for example, sheep red blood cells (SRBC), platelet-rich plasma (PRP), *Pasteurella multocida* and *Bordetella bronchiseptica* as well as VEGF-C as a growth factor. For all, except SRBC, an improved LN regeneration with normal compartment structures was detected, looking at early time points after transplantation [11, 18, 19, 33].

Different treatment strategies of LN prior to transplantation have also been investigated. Goldsmith et al. treated LN with 45% CO_2_ in the air or with normal air but in culture medium for 24 hours and compared them with untreated LN which were directly transplanted after dissection. In addition, human LN, previously frozen and stored in liquid nitrogen, were transplanted into dorsal skin behind the ear in SCID mice. Four weeks after transplantation, the compartment structure and the blood and lymph supply were analyzed. However, all these variations produced no differences in LN behavior [23, 34].

## 6. Differences between LN

**Figure 3:**
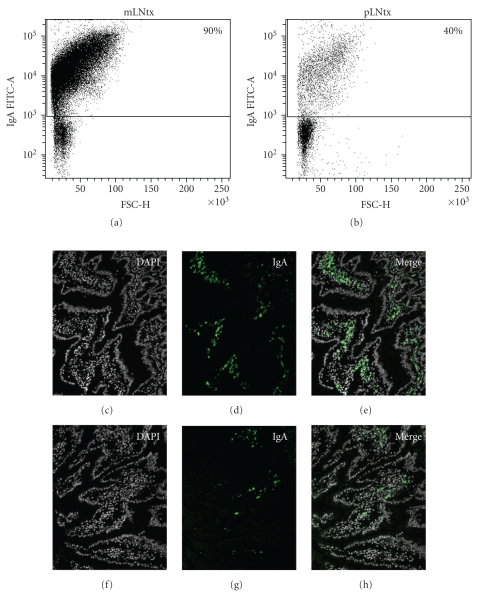
The number of immunoglobulin A (IgA)^+^ cells in the lamina propria is decreased after transplantion of pLN into the mesentery. The gut of mLNtx and pLNtx transplanted animals was analyzed by gating on IgA^+^ cells by flow cytometry. Dot plots of the IgA^+^ cells of mLNtx and pLNtx are shown. Furthermore, immunofluorescence staining of the lamina propria of the gut in mLN transplanted and pLN transplanted rats was carried out with antibodies against IgA (green). Dapi was used to visualize all cells. IgA^+^ cells were seen in both groups, but to a lesser extent in pLNtx animals.

As described above, LN from diverse draining areas showed various differences concerning their homing properties, cell subset appearance, or cytokine pattern. Interestingly, we found many differences in transplanted pLN compared to mLN. For example, after regeneration, pLN transplanted into the mesentery showed neither MAdCAM-1 staining, RALDH2 expression nor the induction of CCR9 or *α*4*β*7 integrin [29, 30]. The lack of these homing molecules (CCR9 and *α*4*β*7 integrin) in pLNtx led to an inadequate induction of a specific immune response in the gut, which is normally induced in the mLN [29, 43]. We detected reduced IgA^+^ cells (Figure 3). After applying cholera toxin (CT) to transplanted animals, reduced CT-specific IgA were observed in the transplanted pLN and also in the gut [29]. Thus, we could show that the draining area has little influence on the microenvironment of LN, and for the first time we identified the stromal cells as an important cell type responsible for the site-specific milieu within the LN.

These first findings were verified by Molenaar et al. who found *α*4*β*7 integrin induction on Ag-specific T cells in mLNtx but no expression on T cells activated in transplanted pLN. Subsequently, they isolated stromal cells which seem to be responsible for the induction and cocultured them with Ag-specific T cells in the presence of or without DC. Here, they were able to show the potential of stromal cells to activate T cells by themselves and of DC to boost this activation [13]. Furthermore, using adult as well as neonatal mLN and pLN for transplantation, it was shown that MAdCAM-1 is usually expressed in mLN, whereas pLN transplants did not show any MAdCAM-1 staining [12, 29]. Thus, the differentiation of the HEV occurs during organogenesis and cannot be altered by transplantation into another draining area.

Furthermore, a further function of the mLN is the induction of oral tolerance. Oral tolerance is the unresponsiveness of the immune system on recognizing a harmless Ag. This phenomenon has rarely been studied and is not understood. Wolvers et al. showed that after transplantation of a pLN in the draining area of the nose (after removing the cLN), tolerance was not inducible [27]. They tolerized the mice on three consecutive days with following immunization and found no reduction of ear thickness in pLN-transplanted mice [27]. Interestingly, we found that mice which received a pLN were more efficient in inducing oral tolerance compared to mLN. We demonstrated that mLNs induce tolerance via the induction of regulatory T cells, which suppress an immune response, whereas pLN induce an immune response via Ag-specific IgG-producing cells, which results in a tolerogenic phenotype [31]. For the first time, we could show differences in the kind of response induction between mLN and pLN. These differences in the induction of tolerance seem to be initiated by stromal cells which maintain their site-specific behavior after transplantation. Thus, the stromal cells of the LN and therefore the microenvironment have a high impact on the induction of tolerance.

## 7. Conclusion and Future Perspectives

The role of LN in the body is not yet completely understood. There are many open questions about the function and the differences between LN and the role of LN within the systemic organisation. Furthermore, the role of stromal cells as a central cell population within the LN has to be elucidated. In addition, all cell types (stromal cells, lymphocytes, and DCs) involved in the induction of an immune responses or tolerance concerning foreign Ag or self-Ag have to be studied in more details individually but much more important in combination with each other. This could be done by transplanting LN into different draining areas. Therefore, LN transplantation is an important method to examine all these questions. Furthermore, the therapeutic advantages of LN transplantation have to be determined in more details.
